# 275. Clinical and Laboratory Predictors of Stroke Associated with COVID-19 Disease

**DOI:** 10.1093/ofid/ofab466.477

**Published:** 2021-12-04

**Authors:** Valerie R Bromberg, Leigh Cressman, Pam C Tolomeo, Hatem Abdallah, Alexis Holmes, Jay Garcia, Ashini Patel, Matthew J Ziegler, Jacqueline Omorogbe, Keith W Hamilton, Ebbing Lautenbach, Michael Z David, Brendan Kelly

**Affiliations:** 1 University of Pennsylvania, Wynnewood, Pennsylvania; 2 University of Pennsylvania School of Medicine, Philadelphia, Pennsylvania; 3 Hospital of the University of Pennsylvania, Philadelphia, PA

## Abstract

**Background:**

Although SARS-CoV-2 predominantly targets the respiratory system, it has also been associated with vascular complications including stroke. Identifying COVID-19 patients at elevated risk for stroke can help inform target anticoagulation strategies. We sought to understand how symptoms and laboratory markers at presentation with COVID-19 relate to stroke risk.

**Methods:**

We enrolled a cohort of 1324 subjects who were hospitalized with COVID-19 across six PennMedicine hospitals between April and August 2020 and performed retrospective, manual chart review to measure exposures including presenting symptoms and admission inflammatory markers. Data were organized with a REDCap database, and analyses were performed using R statistical software, with Bayesian binomial regression models fit using Stan Hamiltonian Monte Carlo via the “brms” package.

**Results:**

Among 1324 subjects, 19 stroke events were observed within 30 days of COVID-19 diagnosis. Admission inflammatory markers, including C-reactive protein (CRP), erythrocyte sedimentation rate (ESR), ferritin, and D-dimer, were poor predictors of stroke risk. Among presenting symptoms, including respiratory, gastrointestinal, dermatologic, and neurologic features of COVID-19 disease, only altered mental status documented on presentation (in 529 subjects) was significantly associated with stroke risk (odds ratio 6.06, 95% credible interval 2.16 - 18.7).

**Conclusion:**

Inflammatory markers associated with COVID-19 disease severity did not discriminate patients at high versus low risk of stroke in this cohort. Altered mental status documented on presentation was significantly associated with incident stroke during COVID-19 disease.

**Disclosures:**

**Ebbing Lautenbach, MD, MPH, MSCE**, **Merck** (Other Financial or Material Support, Member of Data and Safety Monitoring Board (DSMB)) **Michael Z. David, MD PhD**, **GSK** (Board Member)

